# Supplementary material for: Structure of a DNA polymerase abortive complex with the 8OG:dA base pair at the primer terminus
Source: Commun Biol. 2020 Jul 3;3:348. doi: 10.1038/s42003-020-1080-4 (PMC7334213; doi:10.1038/s42003-020-1080-4)
Supplement: Supplementary file 1 — Supplementary Information [file 42003_2020_1080_MOESM1_ESM.pdf]

Supplementary Figures

Supplementary Figure 1

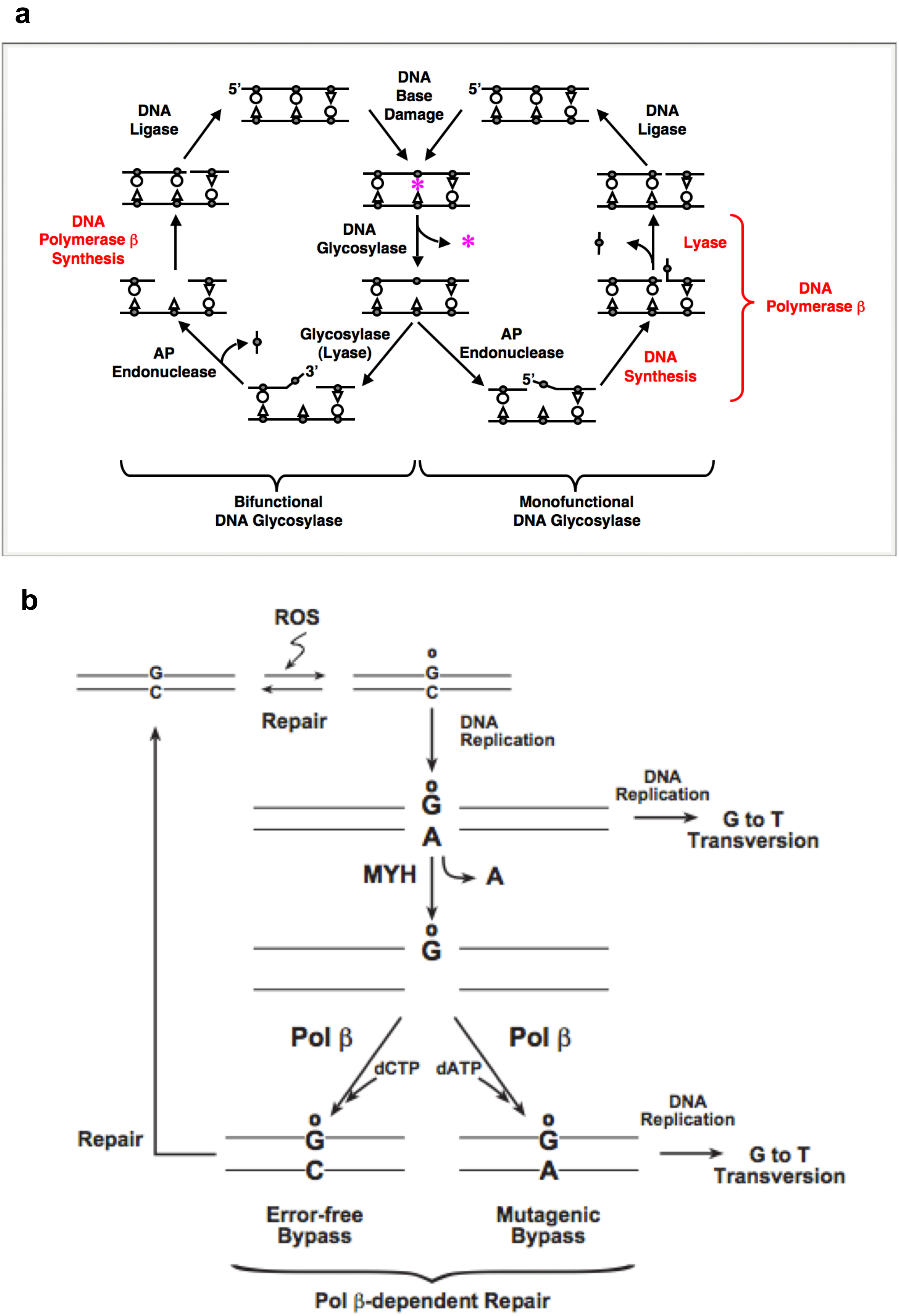

Supplementary Figure 1.
a. Schematics of OGG1 mediated base-excision repair (BER) pathway for the removal of oxidized guanosine (8OG).
b. Schematics of MUTYH mediated base-excision repair (BER) pathway for the removal of dA.

Supplementary Figure 2

a

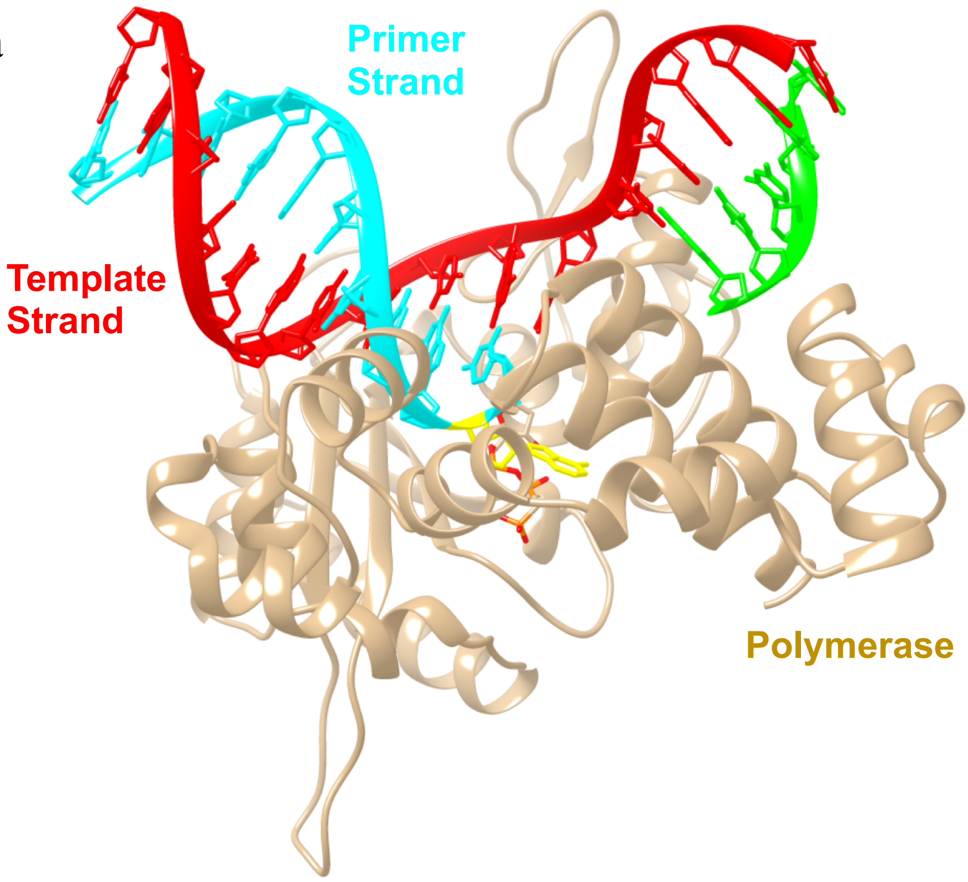

b

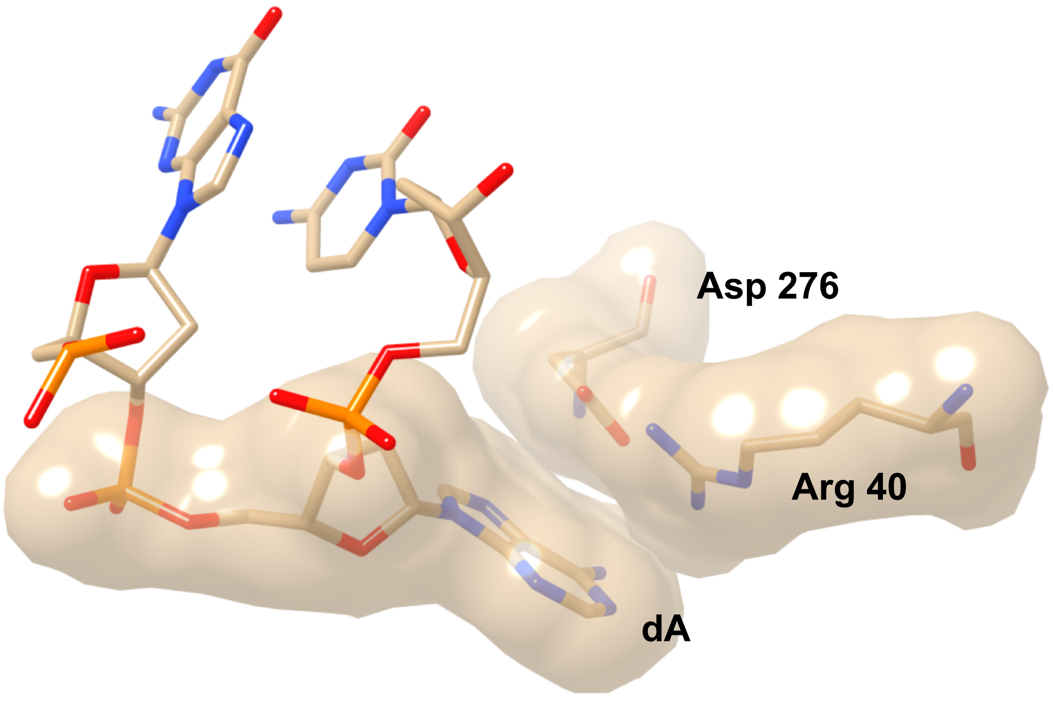

c

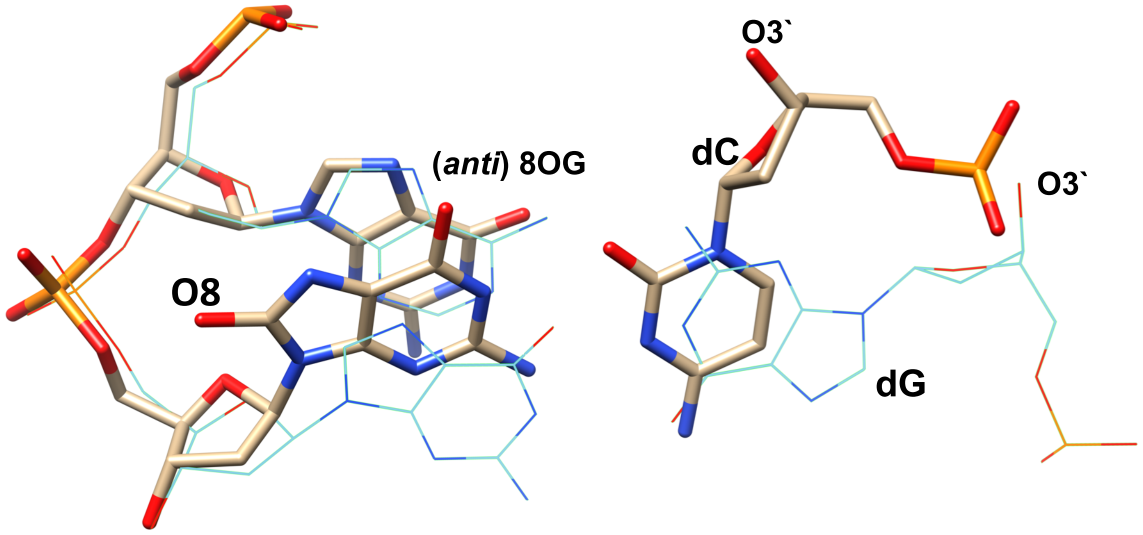

Supplementary Figure 2.

a. Closed polymerase conformation
b. Stabilization of the extra-helical dA base by Arg40 and Asp276
c. Altered polarities of the 8OG: dC and G: G mismatch at the primer terminus
